# Enhanced control and production rates for a green continuous flow synthesis of magnetite nanoparticles: a comparative study of ethylenediamine additives

**DOI:** 10.1039/d5na00773a

**Published:** 2025-10-15

**Authors:** Georgina Zimbitas, Laura Norfolk, Jan Sefcik, Sarah Staniland

**Affiliations:** a Faculty of Medicine, Health, & Social Care, Canterbury Christ Church University Canterbury CT1 1QU UK; b Department of Chemistry, The University of Sheffield Sheffield S3 7HF UK S.S.Staniland@Sheffield.ac.uk Sarah.Staniland@dsit.gov.uk; c Department of Chemical & Process Engineering, University of Strathclyde Glasgow G1 1XJ UK

## Abstract

Scalable synthesis of precise magnetite nanoparticles (MNPs) with controlled properties remains a key challenge for applications in biomedical technologies, data storage, and environmental remediation. Bioinspired additive-driven methods offer greener, tunable synthesis routes, but often suffer from low production rates and limited scalability. Here we demonstrate that our green co-precipitation synthesis with ethylenediamine additives, using continuous static mixing, offers exceptional particle control along with a staggering theoretical production rate of up to 311 g per day, representing a fivefold increase over previously reported methods. This study presents a comprehensive comparison of five ethylenediamine-based additives — EDA, DETA, TETA, TEPA, and PEHA — across three systems: batch, millifluidic, and continuous static mixing. All five additives robustly enhanced octahedral particle morphology (38–84% faceted), compared to the control (no additive, 32% faceted), with a longer chain additive showing the greatest morphological control. These results suggest favourable binding of ethylenediamines to the [111] face of magnetite. This inherently scalable system offers a viable path to industrial-scale, shape-tuned MNP production. TEPA emerged as a standout additive, later refined in a Design of Experiments (DoE) study. While that study focused on a single system, our broader screening establishes critical parameters across additives and synthesis modes, laying the foundation for future optimisation of green, scalable MNP synthesis.

## Abstract 2

Ethylenediamine additives, particularly TEPA, in continuous flow synthesis enhances magnetite nanoparticles formation, realising scalable, sustainable production of green nanomaterials.

## Graphical abstract

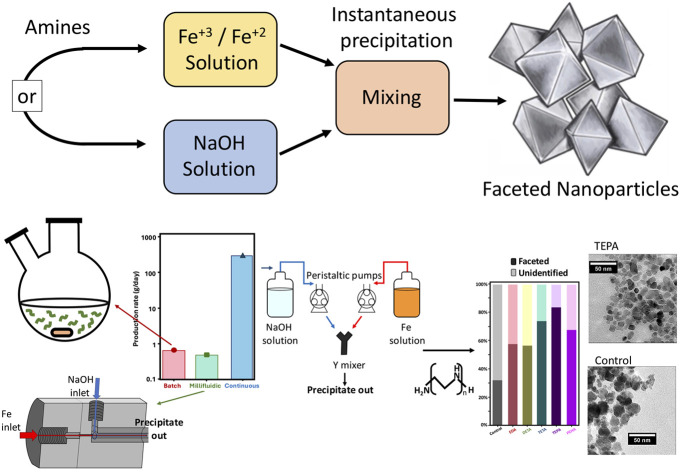

## 1. Introduction

Nanoparticle production is a huge industry, with the global market share estimated to surpass $180 billion by 2028,^1^ and demand for magnetic nanoparticles (MNPs) in particular has recently garnered significant attention, as they have applications in areas including ferrofluids,^2,3^ carbon capture,^4^ high-density data storage,^5^ magnetic drug delivery,^6^ magnetic resonance imaging,^7–9^ and as therapeutic agents in cancer treatment.^10,11^ Critical to these applications is the ability to tailor the size, morphology, and magnetic characteristics of the particles, which directly influence their performance *in vivo* and in technological systems. In addition, as demand grows, so does the necessity for environmentally sustainable and green manufacturing processes. MNPs are widely adapted for use through coatings or surface functionalisation, to be water-soluble,^12^ highly biocompatible, or biodegradable depending on the coating used.^13^ Spherical nanocrystalline magnetite (Fe_3_O_4_) and maghemite (γ-Fe_2_O_3_) particles with diameters less than 20 nm exhibit superparamagnetic behaviour wherein their magnetisation can randomly flip direction due to thermal energy, and as such have been termed SPIONs (superparamagnetic iron oxide nanoparticles). Therefore SPIONs do not have remnant magnetisation in the absence of a magnetic field (unlike larger particles) with the particles instead behaving as if they were paramagnetic.^2^

This “switchable” magnetism is especially attractive in nanomedicine as it allows for MNPs to be magnetically directed to desired sites (such as tumours).^6^ Once the field is removed the particles are less likely to agglomerate, increasing their half-life in the human body.^14^

Many synthetic methodologies and biomedical studies have focused on the use of spherical SPIONs as these are easily synthesised. However, varying the shape leads to different biocompatibility and magnetic properties. A wide research interest in the prospective capabilities of highly faceted SPIONs has recently seen rapid growth of literature involving the use of non-spherical particles.^15–17^ For example, octahedral magnetite particles have been found to exhibit an enhanced specific absorption rate for magnetic hyperthermia and increased performance as MRI contrast agents compared to spherical particles.^18^ It is thus crucial to develop techniques to synthesise non-spherical particles, and to tailor both shape and size of particles to create bespoke SPIONs for specific applications.

What is also of significance is the pressing need for alternative green synthetic production routes that can be industrially scaled-up. Traditional synthesis methods include oxidative precipitation,^19^ hydrothermal synthesis,^20^ and thermal decomposition,^21^ and can produce highly defined particles with specific shapes such as cubic,^22–24^ nanoflowers,^16,24^ and octahedral,^22,24^ however most techniques utilise toxic precursors, large quantities of organic solvents, and/or extensive heating and vacuum use, making such SPION manufacture challenging for environmental compliance.

A simple, room temperature co-precipitation (RTCP) method is an aqueous technique that requires no organic solvents. The pH of a mixed valence iron salt solution is slowly raised by addition of a base, resulting in the precipitation of magnetite (and potentially other iron oxides), all under ambient green conditions. A pH titration study of the RTCP system revealed a strong dependence on the ferric ratio, with lower ratios producing higher proportions of non-magnetite iron oxides.^25^ This process is difficult to finely control, with small variations in reaction conditions resulting in significant changes to the reaction products, such as formation of a broad distribution of SPIONs being of mostly undefined morphology.^26^ So, while this route is both scalable and environmentally compliant, the precision, non-spherical morphological control and reproducibility required is lost.

A promising way to exert control over particle formation in this green synthesis regime is to use an additive. Capping ligands as additives have long been known to increase faceting control of the SPION product and are commonly used in nanomaterial syntheses,^24,27^ as they also provide some control over the particle size distribution of the forming nanoparticles. Such control over nanoparticle shape and size distribution has been achieved in nature with the assistance of proteins. In general, bound proteins or additives can lower the surface energy of a developing crystal face, stabilising that surface and slowing its growth, resulting in it dominating the final particle morphology.^28^ Our previous studies reveal the success of protein-additive RTCP green synthesis with marked increase in morphological and phase/size control over particles formed *in vitro*.^28–30^ Whilst several proteins and peptides have shown promise as biological additives for sustainable nanoparticle synthesis, proteins are expensive and time-consuming to produce and purify, making them unsuitable for scale-up. The concept of additives for green synthesis can be extended to simpler molecules – that are not only readily purchased or synthesised, but are also more compatible with scale-up – to develop a range of bioinspired additives containing functionalities found to be highly effective in nature.

**Fig. 1:**
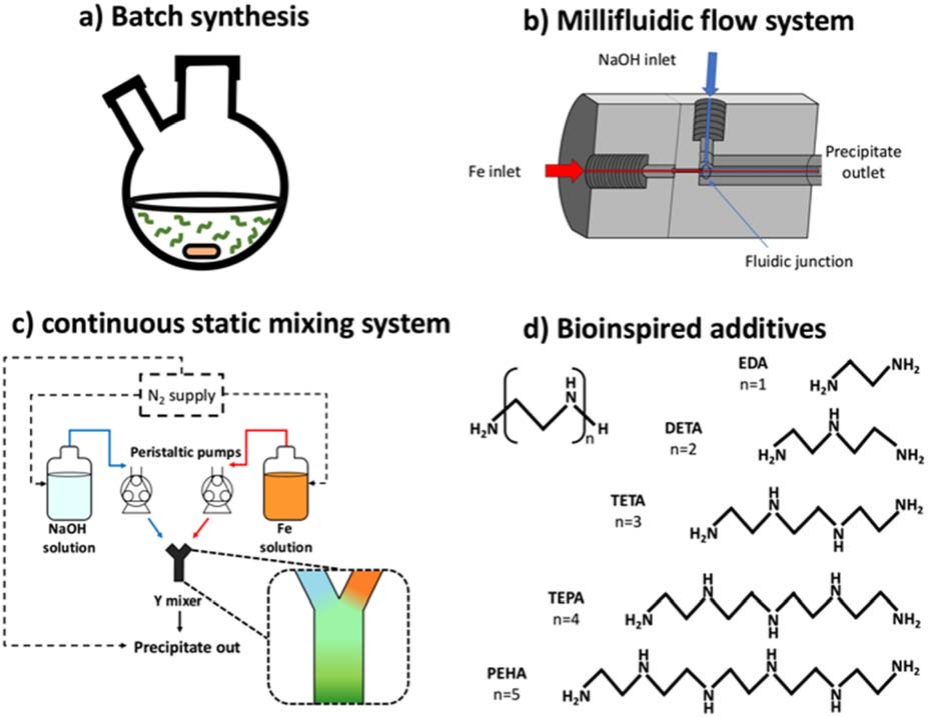
Schematic representations of set-ups used to demonstrate the concept of developing progressive “green” magnetite synthesis systems: (a) Optimised batch RTCP synthesis of magnetite with and without bioinspired additives, (b) millifluidic flow synthesis method,^26^ (c) continuous static mixing set-up, including 2 pumps, 2 feed solutions, a collected precipitate solution, and a Y-mixer. Supply routes are designated with arrows: feed and collected routes are solid lines and N_2_ gas supply in dashed lines, (d) abbreviated names and chemical structures of the bioinspired additive ethylenediamine series used.

With knowledge that amine-rich lysine proteins bind strongly to magnetite surfaces to control the morphology,^28^ we investigated a green synthesis route of MNP production utilising bioinspired ethylenediamine-based compounds consisting of amine groups on an aliphatic –CH_2_ CH_2_– backbone (Fig. 1d), with the series being: ethylenediamine (EDA), diethylenetriamine (DETA), triethylenetetramine (TETA), tetraethylenepentamine (TEPA), and pentaethylenehexamine (PEHA). The series increases in length by one ethylenediamine unit per molecules (Fig. 1d).

Several ethylenediamine-based compounds have shown potential for influencing MNP shape, and in a batch RTCP environment these amines were found to significantly increase the proportion of octahedral/faceted particles formed.^31^ Whereas the use of such compounds appears promising, systematic comparisons across additive types, concentrations, and flow conditions remain rare. Moreover, production rates using batch RTCP have typically remained low, hindering their industrial applicability.

Batch manufacturing often relies on simpler, widely available equipment, however it is continuous manufacturing that is highly attractive for ensuring reproducibility, consistency, and precision in achieving critical quality attributes of particulate products under steady-state operating conditions.^32–34^ Several synthetic methodologies for making magnetic nanoparticles have been implemented using continuous processing, including ambient temperature precipitation,^35–38^ hydrothermal synthesis,^35^ thermal decomposition,^39^ oxidative precipitation,^40^ and high temperature precipitation^41^ in the presence of surfactants. Fluidic synthesis has been utilised for SPIONs on the small scale, with maghemite^42^ and magnetite^26,43,44^ particles both being synthesised on a millifluidic scale (Fig. 1b).

The key challenge in scaling up RTCP processes is to increase the space-time-yield of the overall process, meaning the same end-product quality needs to be achieved in a much shorter reaction time comparable to residence time, noting that the reaction continues in the collection vessel at room temperature residence times. This, in turn, requires much more efficient mixing, which is typically a rate limiting step in precipitation processes. Very efficient mixing can be achieved under certain continuous flow conditions using static mixers,^45,46^ with mixing times on the order of 10–100 ms at typical flow conditions. Scale-up of batch and continuous flow both come with their own problems, but in general batch process scale-up is often fraught with difficulties due to heat and mass transfer issues, whereas by comparison continuous static mixing scale-up is inherently scalable in terms of flow rates, allowing relatively straightforward scale-up towards industrial scale manufacturing.

In this study, we present a comprehensive screening of five ethylenediamine-derived additives – EDA, DETA, TETA, TEPA, PEHA – under three different synthesis conditions: batch RTCP (stirred flask), millifluidic (laminar, coaxial flow in narrow channels), and a novel continuous static mixing system. Green RTCP additive-enhanced synthesis is employed using a continuous static mixing set-up, resulting in the production rate of SPIONs orders of magnitude higher compared to batch and millifluidic systems, while achieving the same end-product quality. Specifically, using the continuous static mixer under laboratory conditions resulted in theoretical yields of up to 311 g per day. Unlike other RTCP additive-enhanced procedures, the RTCP synthesis described in this paper can be characterised as “green” as the process is performed in ambient conditions so does not require heating, toxic organic solvents are not employed, and minute amounts of additives are used. Our unique approach is used to push the boundaries of SPION production rates more than five times higher than reported in the previous literature. Our continuous static mixing approach can facilitate further scale-up for industrial production of tailored faceted SPIONs.

This work establishes key synthetic parameters influencing MNP morphology and magnetic properties, and reveals that longer-chain additives provide superior morphological control, likely *via* preferential binding to magnetite's [111] crystal faces.

These findings also provide a foundational dataset for future optimisation strategies. Notably, building upon the results presented here, there was a subsequent Design of Experiments optimisation focused on TEPA alone,^47^ which validated several key trends identified in our broader screening. In that study, lower magnetism was observed at lower Fe^3+^/Fe^2+^ ratios, whereas stoichiometric ratios close to 0.6 generally produced particles with the highest magnetic properties. In addition, Fe/TEPA ratios of 59 : 1 and 50 : 1, combined with Fe^3+^/Fe^2+^ ratios of 0.59 and 0.6 respectively, resulted in optimal isotropic faceted particle formation, thereby further highlighting the significance of a ferric ratio close to 0.6.

## 2. Materials and methods

Unless otherwise stated, all experimental procedures, reagent sources, and characterisation techniques are as described in our previous studies.^26,31^ Detailed optimisation of the TEPA-based RTCP synthesis conditions has since been published separately,^47^ and only a general overview is provided here for continuity.

Ultrapure MilliQ water (Merck MilliQ integral purification system) was used. All reagents were purchased from Sigma Aldrich, unless otherwise stated, and used as purchased.

In all 3 systems described in this paper additives were premixed into the relevant solution (either Fe precursor or NaOH) prior to loading into the apparatus or, in the case of batch synthesis, prior to NaOH addition. This was done to ensure the additive was already present once mixing had commenced. All solutions were prepared immediately before use and handled under N_2_ to minimise oxidation. Reservoirs remained sealed throughout operation. No post-injection of additives was performed.

### 2.1 Batch room temperature co-precipitation

Optimisation of the TEPA RTCP system, including precise tuning of the Fe^3+^/Fe^2+^ ratio and additive concentration, was conducted separately using a Design of Experiments approach and has been published in full.^47^ In this study, the TEPA data included reflects the initial screening conditions used across all five additives for comparative purposes.

A solution of ferrous sulphate (0.4 mmol) and ferric sulphate (0.3 mmol) was made *via* addition of salts to deoxygenated MilliQ water (20 mL) that had been sparged with N_2_ for at least 30 minutes in a two-neck round bottom flask. This is referred to as the 0.6 ferric ratio solution, previously established as the optimal ratio for magnetite formation due to its proximity to the stoichiometric ratio of ferric and ferrous iron in Fe_3_O_4_ (2 : 1).^26^ The solution was left to stir for 5 minutes using magnetic stirring to ensure dissolution of the iron salts. 500 mmol NaOH (8 mL) sparged with N_2_ was then added at a rate of 50 μL min^−1^ for a total of 160 minutes using a Harvard Apparatus 11 plus syringe pump driver under an atmosphere of N_2_. The reaction was left to age for an hour under the inert atmosphere, and the reaction mixture was then magnetically separated and washed at least three times with N_2_-sparged MilliQ water to remove any nonmagnetic iron oxide by-products. The particles were dried in a 40 °C oven overnight, and then ground with a pestle and mortar for analysis. A schematic representation for this set-up can be seen in Fig. 1a.

### 2.2 Millifluidic flow

A simple schematic representation of the set-up can be seen in Fig. 1b. For the full synthetic methodology and device fabrication details please refer to Norfolk *et al.*^26^. Briefly, it is a co-axial fluidic device with a fluidic junction between the two streams of reactants. The Fe precursor solution is introduced through the central capillary (inner, core flow), while the NaOH solution is introduced through the surrounding channel (sheath flow), resulting in a focused core stream. This configuration promotes rapid mixing and particle formation downstream while reducing clogging at the inlet. The device was cleaned prior to use with 500 mM HCl and then with MilliQ water for several minutes. A solution of 50 mM mixed valence (ferrous and ferric sulphate) iron salts with a 0.6 ferric ratio, and a solution of 1 M NaOH were both deoxygenated *via* sparging with N_2_ for a minimum of 30 minutes. A Harvard Apparatus 11 plus syringe pump driver was connected to the outer sheath flow inlet loaded with a 10 mL Luer lock syringe of NaOH and connected to a millimetric co-axial fluidic device *via* polyether ether ketone (PEEK) capillary tubing. This outer flow was set at a flow rate of 360 μL min^−1^. A second syringe pump driver was connected to the inner core flow and loaded with a 10 mL Luer lock syringe filled with 8 mL of 0.6 ferric ratio Fe salt solution and connected *via* PEEK capillary tubing. This was set at a flow rate of 90 μL min^−1^. The NaOH syringe was refilled as required. The total flow was taken as (90 + 360)μL min^−1^ = 450 μL min^−1^ through 0.508 mm inner diameter PEEK tubing, thus producing a channel Reynolds number of approximately *R*_e_ ≈ 18 for a water solution at 18 °C. This confirms strictly laminar flow and mixing that arises from coaxial focusing, rather than turbulence. Taking under consideration the fact that millifluidic systems are prone to fouling from clogging, with this being exacerbated in a system producing magnetic nanoparticles, precautions were taken to minimise this effect. Issues were addressed by selecting a specific diameter of tubing, ensuring a straight fluidic channel, and adjusting the needle used to cast the device so as to ensure a smoother co-axial junction.^26^

The iron oxide material formed and flowed to the end of the device where it reached the exit port and dripped into a round bottom flask which was kept under an atmosphere of N_2_. The iron oxide product was magnetically separated and washed three times with deoxygenated MilliQ water and subsequently dried in a vacuum oven at 40 °C overnight. The particles were then ground with a pestle and mortar for analysis.

### 2.3 Continuous static mixing

As in the case of the millifluidic flow system, the Fe solutions used here were of a ferric ratio of 0.6. In addition, a total iron concentration of 50 mM and NaOH concentration of 500 mM were used, to remain consistent with previously conducted research.^31^ Two Watson Marlow 520DuN (Zwijnaarde, Belgium) cased peristaltic pumps were used, with one attached to the NaOH feed and the other attached to the Fe solution feed. Mixing and instantaneous precipitation both occur in a plastic Y-shaped static mixer which was connected to an outlet tube leading to the collection vessel. Both pumps were set to 10 rounds per minute, averaging approximately 112 mL min^−1^. When both pumps were running concurrently this setup resulted in a residence time of approximately 2 s for the mixed solution in the outlet tube.

Fe solution was fed through one pump, whereas NaOH solution was co-currently fed through the other pump (Fig. 1c) in a 1 : 1 volumetric flow ratio. Feed solutions each contained a magnetic stirrer bar, with the solutions placed on magnetic plates to be under constant mild stirring throughout the experimental run. All solutions (feeds and product output) were constantly supplied with N_2_ throughout the run to ensure the solutions were sparged of oxygen, thus minimising the possibility of unwanted oxidation occurring at any stage of the precipitation process. The feed solutions were left to stir for at least 5 minutes under a constant atmosphere of N_2_ prior to mixing, to ensure deoxygenation was complete.

Modified lids were used to avoid the re-dissolution of O_2_ into any of the three solutions (Fe solution stock, NaOH stock, collection vessel) during the experimental runs. These modified lids incorporated 3 openings: one for the pump feed tubing, one for the N_2_ supply, and the third with tubing that allowed for gas/pressure to escape the vessel. Once the initial 5 minutes of stirring of the feed solutions was complete, both peristaltic pumps were turned on (counterclockwise flow) for mixing to occur. Mixed solution was collected only after the flow became constant. Timing the collection of 15–20 mL of mixed solution resulted in a calculated flow rate of 110–120 mL min^−1^ from both pumps combined, with an average of 112 mL min^−1^ across the 31 experiments. At the flow rates used (approximately 2 mL s^−1^ with inner tube diameter of 2 mm), and assuming room temperature of 18 °C, the Reynolds number was calculated as being approximately 1200, well below the threshold for turbulence (*R*_e_ > 4000), identifying the system as operating under laminar conditions with mixing efficiency determined by the static mixing elements rather than turbulence.

Once mixing was initiated, the system would operate for short durations (up to 90 s per run), after which the lines were flushed to prevent cross-contamination and to remove deposited material. This occurred between all precipitation runs. Visual inspection revealed some build-up of precipitate within the Y-mixer and outlet tubing, consistent with the tendency of such designs to foul during nanoparticle precipitation. As such, the system was flushed out with in-house ultrapure MilliQ water until the fluid ran clear from the collection tube. The flow was then reversed on both pumps (clockwise) and MilliQ water was then left to flow out of the feeding tubes, again, until the water ran clear.

32 experiments are included in this study with effects of the amine additive, additive-to-total iron ratio, and additive addition point. Each additive was tested at three different additive : total iron ratios: 1 : 100, 1 : 1000 and 1 : 10 000. The additive was introduced *via* either the Fe solution or NaOH inlet (referred to as addition points), resulting in a total of six experiments for each additive.

The full list of conditions can be seen in Table 1, with the amines used being TEPA (experiments 2–7), TETA (experiments 8–13), PEHA (experiments 14–19), DETA (experiments 20–25) and EDA (experiments 26–31). A control experiment, whereby no additive was used, was also performed for comparison (experiment 1). The collected particles were magnetically separated, washed three times with deoxygenated MilliQ water, and dried in a vacuum oven at 40 °C overnight. The particles were then ground with a pestle and mortar for analysis.

**Table 1:** Additives and additive : iron ion molar ratios used for magnetite precipitation with 500 mmol NaOH and 50 mmol total Fe at a 0.6 ferric ratio

Additive : iron ion	1 : 100	1 : 1000	1 : 10 000	Additive added to:
Additive	Number of experiment			
No additive	1			N/A
TEPA	2	3	4	50 mM Fe
	5	6	7	500 mM NaOH
TETA	8	9	10	50 mM Fe
	11	12	13	500 mM NaOH
PEHA	14	15	16	50 mM Fe
	17	18	19	500 mM NaOH
DETA	20	21	22	50 mM Fe
	23	24	25	500 mM NaOH
EDA	26	27	28	50 mM Fe
	29	30	31	500 mM NaOH

### 2.4 Characterisation

#### 2.4.1 Transmission electron microscope (TEM)

For sample analysis of magnetic nanoparticles, a 1 mg mL^−1^ suspension of nanoparticles was sonicated for 1 minute in hexane, after which a 10 μL sample was dropped onto a carbon coated copper TEM grid and allowed to dry down for a minimum of one hour. Grids were imaged using a FEI Tecnai G2 Spirit electron microscope (Thermo Scientific, Waltham, MA, United States) and discrete particles of the TEM images were analysed using ImageJ software (v1.52, public domain, National Institute of Health, MD, USA). For each sample a minimum of 200 particles were randomly selected for measurement. TEM image analysis for particle size and shape was performed with the aid of ImageJ software *via* the method described in Norfolk *et al.*^31^ The methodology used is often employed, however it is subject to a degree of error. This, coupled with the fact that the magnetic nanoparticles would agglomerate, means shape and size analysis *via* TEM alone is difficult and as such the results presented here are approximations that do, nonetheless, allow for comparisons and indications of observable trends between the samples presented in this paper.

#### 2.4.2 X-ray diffraction (XRD)

XRD data of samples was collected by analysis of dry iron oxide nanoparticles in a Bruker D8 powder diffractometer (Bruker, Coventry, United Kingdom). Diffraction images were collected at 0.022-degree increments from 20–80°, using a fixed wavelength of *λ* = 1.54178 Å from a Cu Kα X-ray source. Whereas some impurities may be present (*e.g.*, due to undesirable oxidation occurring prior to XRD measurements), the particles examined were black, indicating magnetite and not maghemite, the latter of which has brown coloured nanoparticles. In addition, the iron solutions used were of a ratio that strongly favours the formation of magnetite. Therefore, the presence of maghemite cannot be excluded, but is also not significant enough to influence the overall results.

Reference lines for spinel (magnetite/maghemite), wüstite (FeO), and hematite (α-Fe_2_O_3_) have been overlaid in the XRD patterns of Fig. 3d, and are represented as M, W, and H respectively. Numbers in brackets refer to the crystal plane of the respective peak. The figure reveals that despite efforts to avoid oxidation it can occur to a small degree, and this appears to be the case for the control and TEPA samples shown in the figure. The other samples lacked FeO and hematite features, indicating oxidation had indeed been avoided as they mainly consisted of peaks for the expected spinal product.

#### 2.4.3 Vibrating sample magnetometry (VSM)

Magnetic susceptibility and saturation magnetisation were measured on a known quantity of dry iron oxide nanoparticles. Specifically, 1–5 mg of accurately weighed powder precipitate was examined using a MPMS 3 SQUID magnetometer (Quantum Design, Surrey, United Kingdom) in vibrating sample mode, with the samples packed in size 3 gelatine capsules and immobilised with polytetrafluorethylene (PTFE) tape. The samples were run at 300 K between −3 and 3 T with a sweep rate of 0.01 T s^−1^. The magnetisation result was then linearly scaled to the equivalent of 1 gr of the measured sample. The data presented is cropped at saturation magnetisation for ease of viewing.

## 3 Results & discussion

### 3.1 Comparison of continuous static mixing system with batch RTCP and millifluidic flow systems: scale-up in the absence of additives

Three systems were investigated: batch RTCP, millifluidic flow, and continuous static mixing. The latter two flow systems are passive (static) mixers, meaning that moving parts where not partaking in the mixing: the millifluidic device relies on coaxial laminar sheath flow, while the larger-scale continuous static system uses a static mixer (Y-mixer) for rapid inline mixing. All three systems used the same room-temperature co-precipitation (RTCP) synthesis, whereby the pH of a solution of mixed valence ferrous/ferric iron ions was raised to precipitate out the iron oxide nanoparticles under an inert atmosphere. Varying the ferrous/ferric ratio results in different iron oxides being formed. Magnetite (Fe_3_O_4_) is the desired product and is most readily formed at a ratio of 0.6 ferric ions^26^ which is close to the stoichiometric 2 : 1 ratio of Fe^3+^/Fe^2+^ ions in the structure of magnetite.

In this study a 50 mmol mixed valence iron solution, with the iron species fixed to the 0.6 ferric ratio, was used for all three systems. The concentration of NaOH was set at 500 mmol for the batch RTCP and continuous static mixing set-up, as this was previously optimised for the batch RTCP system.^31^ 500 mmol NaOH resulted in non-magnetic iron oxides in the millifluidic system whereas 1 M NaOH was found to be optimum.^26^ As such, 1 M NaOH was used in the millifluidic system.

Each sample was analysed by TEM imaging to ascertain shape and size of particles formed, XRD for crystallinity and phase identification, and VSM for magnetic data. More information on how particle shape and size were acquired can be found in the SI, under Sections S1and S2. In brief, visual inspection was performed, whereby particles that were clearly faceted were identified as such, whereas remaining particles (*e.g.*, overlapping, blurry, and/or particles of uncertain shape) were classified as unidentified.

Using only magnetically separated material and assuming that a 100% conversion rate of the precursor is achieved, the theoretical production rates for each system were calculated as presented in the SI (Section S12), and are summarised as follows: 0.69 g per day for batch RTCP, 0.50 g per day millifluidic flow system, and 311 g per day for the continuous static mixing system.

**Fig. 2:**
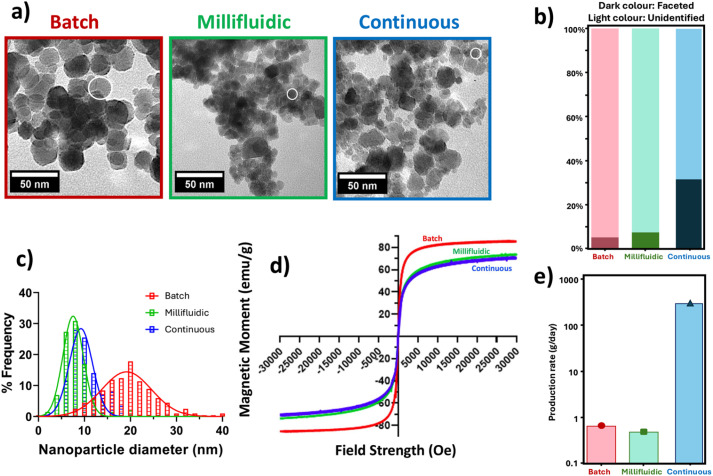
Results for the MNPs formed at the 0.6 ferric ratio. Colour codes used: batch RTCP (red), millifluidic flow (green), and continuous static mixing (blue). (a) representative TEM images. White circles indicate approximate average size of particles, (b) comparison of shape analysis results, (c) particle size frequency distribution, (d) magnetic data, (e) production rate comparison of the three synthetic methods.

For each system, control reactions were carried out to ascertain the morphology and quality of particles formed when no additive was present. The millifluidic 0.6 control reaction has previously been published,^26^ and this reaction has been selected to act as a comparison between the three systems. TEM images of the particles formed across each of these three set-up systems are shown in Fig. 2a. All five ethylenediamine additives significantly influenced particle morphology compared to the no-additive control (Fig. 2b).

While the control condition produced a majority of undefined or spherical particles (32% faceted), the addition of any ethylenediamine increased the proportion of faceted particles to between 38-84%, depending on concentration and chain length. Overall, longer chain additives (TETA, TEPA, PEHA) consistently produced a higher percentage of well-defined octahedral morphologies, indicating a stronger interaction with the [111] crystal face of magnetite. These results align with previous studies showing that basic residues, such as amines, stabilise specific crystal facets *via* selective adsorption.^28^

When compared to the particles of the continuous static mixer, the particles formed in the batch RTCP and millifluidic flow systems were found to exhibit minimal shape control, as shown in Fig. 2b, with undefined particles being the primary constituent of the two reactions. Specifically, the proportion of undefined particles varied from 92% for the millifluidic flow system, to 94% for the batch RTCP systems. The continuous static mixing system exhibited a higher degree of shape control, producing 32% faceted particles. A significant variation was also observed in particle size, as shown in Fig. 2c. The millifluidic flow and continuous static mixing systems produced particle sizes of 8.7 ± 4.1 nm and 9.2 ± 3.6 nm respectively, whereas the batch RTCP system resulted in particle sizes of 19.5 ± 6.2 nm, thus resulting in particles almost double the size of those produced in the other two systems, and with a much broader size distribution. This behaviour is consistent with previous reports showing that flow reactors improve mixing efficiency and supersaturation homogeneity, thereby favouring numerous nucleation events over extensive growth and limiting polydispersity.^26,31,50^ Flow rate also plays an important role, with higher flow rates increasing shear and reducing mixing and residence times, thus further promoting nucleation relative to growth, leading to smaller average particle sizes. Lower flow rates result in less efficient mixing, leading to the formation of larger particles, broader distributions, or even aggregation and fouling.^50^

Results show the millifluidic flow system produced nanoparticles with the narrowest size distribution among the three synthesis modes (Fig. 2c). This is probably due to the stable flow and well-defined residence time achieved in the channel system, which minimised variability in nucleation and growth environments. While the system allowed for continuous processing, the reliance on laminar sheath flow constrained scale-up potential and mixing efficiency. Yields remained low, consistent with previous studies using Mms6-modified particles in millifluidic channels.^26^

Overall faceting trends followed those seen in the batch system, with longer-chain additives yielding a higher proportion of octahedral particles. The millifluidic flow setup consistently delivered more monodisperse particle populations, however, throughput remained limited due to small channel dimensions and low operational flow rates, resulting in theoretical production rates well below those of the static mixer system. These observations confirm the utility of millifluidic processing for producing high-quality, uniform nanoparticles, particularly where low volume and tight size control are critical. Nonetheless, its scale-up potential is restricted due to geometric and flow regime constraints.

Magnetic data gathered from the three systems shows a relatively high magnetic moment (emu g^−1^) of material from each reaction, having values of 85.8 emu g^−1^ (batch RTCP), 73.8 emu g^−1^ (millifluidic flow), and 71.4 emu g^−1^ (continuous static mixing) at a field strength of 3 T, with pure magnetite having a value of 92 emu g^−1^ by comparison. Both millifluidic flow and continuous static mixing produced slightly less magnetic particles than those formed in the batch RTCP system. XRD data (SI, Section S3) for each reaction was found to be consistent with magnetite. The presence of lesser magnetic particles could be due to the oxidation of the nanoparticles. Whereas precautions were taken to minimise the degree of oxidation, the possibility of it occurring remained. For the millifluidic system the reagent solutions were pre-sparged with N_2_. They were then loaded into syringes which are not perfectly airtight, thus possibly allowing a small amount of oxidation to occur throughout the reaction process, leading to the potential of less magnetic particles. In the continuous static mixing system, each stock solution and the resultant mixed solution are consistently sparged with N_2_ throughout the experiment, with the retention time in the system being significantly lower than the time required in the millifluidic flow system due to the disparity in the flow rates. Despite this, the collected nanoparticles remained in solution for an extended period of time as they needed to be transported for analysis, again possibly resulting in a slight degree of oxidation and, subsequently, slightly lower magnetisation compared to the batch RTCP system.

In summary, both millifluidic flow and continuous static mixing systems show a consistency in size of particles produced, being of 10 nm, while batch RTCP synthesis offers particles of 20 nm. When comparing the millifluidic flow system to the continuous static mixing system, the later produces similar highly magnetic magnetite particles but at a far greater production rate than the former, with the promise and potential for further scale-up. Assuming a 100% conversion rate of the precursor and including only magnetically separated material, a theoretical production rate was calculated for all three systems run over a 24-hour period (Fig. 2e and SI, Section S12).

Results show the continuous static mixing system can potentially offer an impressive improvement in particle production capacity, producing over 311 g of bespoke nanoparticles per day (SI, Section S12).

Properties of the nanocrystals are, however, not only associated with their size but also their shape.^48,49^ As such there is a need to achieve a narrow particle size distribution of a uniform shape in order to obtain high-quality nanoparticle production.

### 3.2 Effect of EDA series of additives in the continuous static mixing system

A series of amines (Fig. 1d and Table 1) were selected for use as additives in the continuous static mixing system. DETA-PEHA additives have been shown to influence the morphology of particles formed in RTCP reactions *via* adsorption to the [111] crystal face of developing magnetite nanocrystals producing octahedral nanocrystals. Computational modelling has shown this is due to preferential adsorption to the [111] crystal face (over [100]) of the forming magnetite nanocrystals, increasing its dominance in the final crystal habit. Here we test if such additives can influence the morphology of magnetite nanoparticles formed in the large-scale continuous static mixing system.

Each ethylenediamine additive, from chain length (number of ethylene units) *n* = 1–5 (EDA (*n* = 1); DETA (*n* = 2); TETA (*n* = 3); TEPA (*n* = 4); PEHA (*n* = 5)), was added at various concentrations (additive : iron ratios 1 : 100, 1 : 1000, and 1 : 10 000) and tested with addition in each of the iron and NaOH channel inlets. For consistency with results from the previous section, and so the continuous static mixing results without additives can be used as a control, the reaction conditions were identical to those of previous sections (0.6 ferric ion ratio with iron solution of 50 mmol and NaOH solution of 500 mmol concentration).

**Fig. 3:**
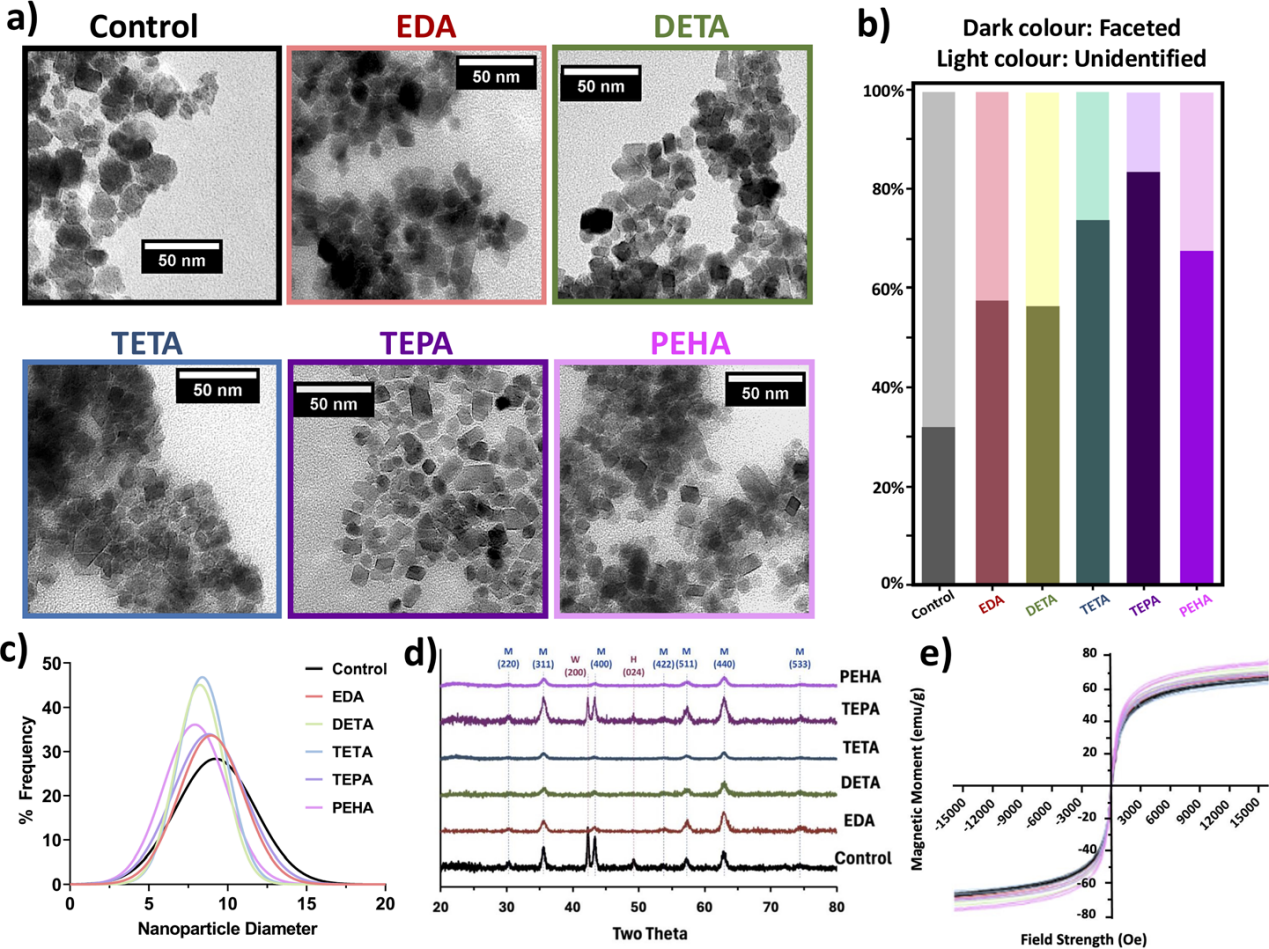
Comparison of effect of additives on magnetite nanoparticles formed with continuous flow mixing at 1 : 100 additive iron ratios (Fe inlet) (coloured) and batch RTCP control (black). (a) TEM images, (b) shape analysis, (c) particle size distribution, (d) XRD, (e) magnetic data.

Fig. 3 shows the results for the particles formed when an additive was added to the iron solution inlet at the highest additive : iron ion ratio, that being 1 : 100. TEM images (Fig. 3a) show the morphology is significantly improved with the addition of any of the selected ethylenediamine-based additives, resulting in clearly more faceted and angular nanoparticles from 32% (control) to 58–84% with additives (Fig. 3b). 2D TEM images were used to distinguish faceted and non-faceted particles, with faceted particles appearing to be cubic, hexagonal, octahedral, and diamond-shaped. It is important to note here that when a 3D shape is visualised in 2D, an octahedral particle most commonly appears to be diamond shaped but can also appear hexagonal or even square if viewed down different axes. As such, TEM images here were not used to assign a specific shape to the particles, but rather to easily distinguish faceted and non-faceted particles. In this case, TEPA presence shows the most control with 84% faceted particles compared to 57% for EDA and 58% for DETA, the latter two of which offer the least control. Fig. 3c shows that compared to the control, particle size is not significantly affected by the addition of the ethylenediamine additives.

These results generally agree with a previous batch study with the same additive series, whereby crystallisation was directed to faceted (octahedral) particles.^30^ Previously, TETA, TEPA, and PEHA were observed to offer excellent morphological control, which is reflected similarly in these results. At this point we must note that the additive presence could not offer quite the same level of morphological control here as that achieved in a batch process (batch process previously produced 1.2× more octahedral/faceted particles relative to each additive),^31^ however the timeframe in which particles were obtained using the continuous static mixer set-up is substantially quicker (seconds, as opposed to hours).

### 3.3 Effect of process conditions in the continuous static mixing system

All additives were used in further studies to investigate the effect of additive concentration and addition points. The ratios of 1 : 100, 1 : 1000, and 1 : 1000 of additive : iron ions were selected to investigate additive effectiveness as well as additive addition location (introduced in either the iron or base reagent solution). The 3 different concentrations with 2 different addition points resulted in 6 different particle samples to analyse for each additive (total of 30 data sets, see SI Fig. S4–S8 for TEM, size and shape analysis, XRD and magnetism for each additive individually).

**Fig. 4:**
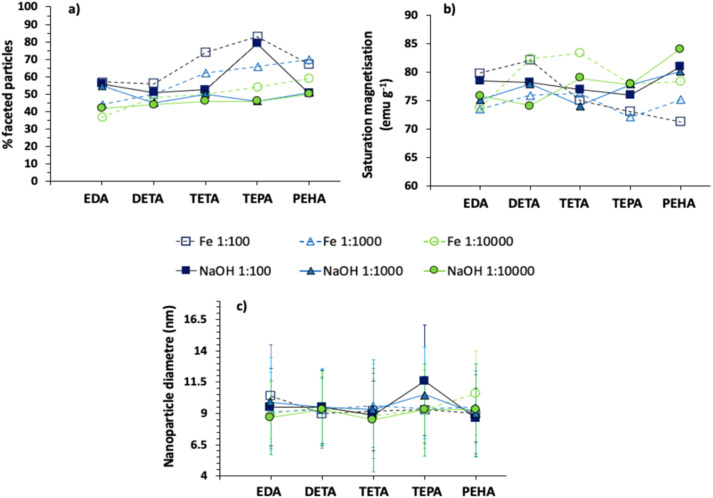
Effect of additive : iron ratios and additive feed (addition to Fe or NaOH inlet) for each additive. (a) Effect on percentage faceted particles, (b) effect on saturation magnetisation, (c) effect on mean particle diameter (more details can be found in the SI, Sections S9–S11). Range bars show highest and lowest values measured. Data from (a) and (b) are from single representative experiments, whereas data from (c) is from samples containing approximately 200 particles.

The addition of additives in any proportion resulted in an increase in proportion of faceted particles observed, with a minimum of 38% and a maximum of 84% being observed (Fig. 4a) compared to 32% in the comparable control. The general trend is that more control is exerted over faceted particle formation using additives at the longer amine lengths.

Prior molecular dynamics simulations of the batch system revealed that reactions with PEHA each exert similarly good control, which was not the case for DETA.^31^ In these simulations DETA shows no preference for either the [111] or [100] face of magnetite, and hence does not as efficiently promote faceted particle morphology. Modelling also showed the longer ethylenediamine additive molecules can bind strongly to the [111] surface, with increased numbers of both Fe–N and O–H interactions.^31^ We propose the same advantage of longer additive chains in the batch system applies for the continuous mixing system presented here. There is, however, an exception to this trend; for the highest concentration of additives (1 : 100) added to either the iron or NaOH solution, we see TEPA has the highest quality (proportion of faceted) particles, followed by a reduction of faceted particles for PEHA (for the same conditions, Fig. 4a). This, again, is very interesting and suggests there is an optimum number of amine groups beyond which control over crystal habit is reduced. At the additive : iron 1 : 100 ratio, the PEHA concentration used will have more amine groups present than the equivalent TEPA used, however the amines PEHA introduces may go beyond the optimum amines required under these conditions. As such, the highest quantity of faceted particles is achieved for PEHA at the middle concentration of 1 : 1000 additive : iron ratio, producing 70% faceted particles compared to 67% at the 1 : 100 ratio.

While the main difference in faceted particle formation is seen by varying additives, there are some trends seen with respect to the reagent inlet the additive is introduced in and the additive : iron ion ratio. In general, introduction of additives *via* the Fe solution inlet resulted in higher percentages of faceted particles produced with respect to introduction *via* the NaOH inlet, under the same conditions, with the difference becoming more evident at longer additive chain lengths. This behaviour could hint to a small advantage of protonating the longer amines, which would only occur in the more acidic iron solution. This could be expected to be attractive to a negatively charged magnetite surface. It is not clear why the neutral EDA in basic conditions would produce more faceted particles than the EDA introduced to the reaction in the acidic iron solution. The continuous static mixing environment is much more effective in homogenising local concentrations compared to more heterogeneous environments in mixed flasks or under laminar flow conditions, so that local concentrations of positive and negative ions are much more uniform.

When TEPA was used at the highest concentration of 1 : 100, a high percentage of faceted particles were produced from use in either inlet (84% with additive added *via* Fe solution compared to 80% *via* NaOH solution). For the medium and lowest additive : iron concentrations (1 : 10 000 and 1 : 1000), faceted particle percentages showed an upward trend with amine chain length from DETA to PEHA, and introduction of additives through the Fe solution inlet showed a higher efficacy to longer chain amine additives than introduction *via* the NaOH solution inlet. TEPA appeared to be highly effective at the 1 : 100 concentration, a lot more so than PEHA.

In general, higher quantities of faceted particles occur with higher concentrations of additives across the full range of additives and regardless of inlet solution (Fig. 4a and SI, Section S9) with the exception of PEHA and TETA 1 : 100 (NaOH inlet), as already discussed. For example, EDA at a 1 : 100 additive : iron ion ratio produces 58% faceted particles compared to 38% at the 1 : 10 000 concentration when added *via* the iron solution, showing approximately 20% difference. Similarly, for EDA added *via* the NaOH solution, the 1 : 100 ratio yields 57% faceted particles *versus* 43% at the 1 : 10 000 ratio, again showing approximately 15–20% difference. There is a much smaller difference with concentration for DETA (insignificant for DETA in the NaOH solution) and a larger difference observed for TETA and TEPA (excluding the TEPA 1 : 100 result discussed prior) (Fig. 4a).

While this trend is consistent it is worth noting that the additive concentration increased by 100-fold between the lowest and highest concentration for only a marginal 15–20% improvement. These marginal improvements in particle shape would not be likely to offset additional costs of using 10- or 100-times larger amounts of additives per unit mass of particles produced, meaning it would appear to be most cost effective to use the lower additive concentration. Furthermore, it is worth highlighting the most faceted SPIONs (70%) are achieved with PEHA at the mid-concentration of 1 : 1000 introduced through the iron ion channel (Fig. 4a), demonstrating that there is a more significant difference seen by varying the additive used rather than varying additive concentration.

The effect of the additive is predominantly confined to altering the particle morphology, with no significant difference observed in magnetism (Fig. 4b and SI, Section S10) or size (Fig. 4c and SI, Section S11) across all additives, concentrations, and addition inlet. The magnetic saturation of all samples lies between 83.5 emu g^−1^ (for PEHA NaOH 1 : 10 000) and 65.8 emu g^−1^ (for PEHA Fe 1 : 100) (SI, Section S10). It is noteworthy that PEHA magnetic saturation is highest (higher than in the control sample) for the lowest concentration of additive in base, with magnetic saturation reducing as the concentration of additive increases, then reducing furthermore with additive added in the iron channel at the lowest to highest concentration, giving the lowest magnetic saturation (Fig. 4b). There is a small increase in magnetic saturation in samples produced with increasing length of additives, added in NaOH, whereas the magnetic saturation of samples produced with additives in the iron solution see a peak at DETA and TETA. The highest saturation quantities are from samples produced with the lowest concentration of additives. There is no difference in size across all particles, in keeping with the data from the batch study and the hypothesis that the amine is not nucleating particles but is solely a shape controlling additive.

Among the five additives screened, TEPA emerged as particularly effective in producing highly faceted, monodisperse particles across all three systems. Its performance under RTCP conditions prompted further optimisation using a Design of Experiments strategy, which has been reported separately.^47^

Range bars shown in Fig. 4c represent the minimum and maximum particle sizes measured from approximately 200 particles per sample, with the majority of particle sizes falling close to the mean, as verified from frequency distributions (see SI, Sections S4–S8).

The results from that study validated several of the trends identified in our broader screening, particularly the influence of Fe/additive ratio and Fe^3+^/Fe^2+^ composition on particle morphology and magnetisation. Specifically, Fe/TEPA ratios of 59 : 1 and 50 : 1, combined with Fe^3+^/Fe^2+^ ratios of 0.59 and 0.6 respectively, were found to yield optimal isotropic faceted particles with enhanced magnetic properties. These findings reinforce the significance of a ferric ratio close to 0.6 and demonstrate how our wide screening dataset can inform predictive optimisation of synthesis conditions.

## 4 Conclusions

Table 2 summarises the results presented in this study. When producing specific iron oxides, fine-tuning and control over particle morphology is readily achieved using thermal decomposition and hydrothermal syntheses, however these methods use excess amounts of energy and often employ organic solvents, making them hazardous to the environment and very costly. Using room-temperature co-precipitation techniques have the downside of producing inconsistent particles with a high degree of polydispersity^28^ and often include the formation of undesired oxides,^25,51^ however they are performed under ambient conditions and usually use aqueous-based solvents, making RTCP energy and cost efficient, but also more environmentally friendly. Current RTCP methods do not produce enough desirable products to be commercially viable, and as such the aim is to increase their productivity. In this paper we have demonstrated that this is possible using a continuous static mixer set-up and utilising the capping agent abilities of specific ethylenediamine additives. Particles formed using RTCP conditions generally exhibit a high degree of polydispersity. Such was the case across the three different systems (batch RTCP, millifluidic flow, and larger-scale continuous static mixing) used in this paper when examining particles formed without the addition of additives. There is poor shape control, with undefined morphologies making up 68% (continuous static mixing) to 94% (batch) of the particles present. The batch system produces particles approximately twice the diameter (20 nm) of those formed in the other two fluidic systems (9–11 nm). Despite this, each system produces highly magnetic particles, with the most magnetic being formed in the batch system. The inclusion of ethylenediamine-based additives significantly affected the morphology of the resultant particles, with the addition of any of the additives increasing the proportion of faceted particles from 6–32% for the controls, to 38–84%. These results suggest additives play a key role in shape direction of the forming iron oxide particles. The addition of additive in any proportion led to a substantial increase in ratio of faceted particles observed, with a general trend of more additive resulting in a higher proportion of faceted particles.

**Table 2:** Summary of results obtained for 3 different systems: batch RTCP (batch), millifluidic flow (millifluidic), and continuous static mixing (cont. static)

Parameter:	Batch	Millifluidic	Cont. Static
Size distribution	Broad	Narrow	Moderate
Faceting	Additive-dependent, variable	Additive-dependent, improved	High, consistent across additives
Productivity	Low	Very low	High (311 g per day)
Scalability	Low	Limited (channel size)	High (flow-rate scalable)
Reproducibility	Variable	Good	High

TEPA showed to be a notable exception, showing more faceted particle control at the high amine : iron ion ratio concentration of 1 : 100. TEPA's performance under RTCP conditions prompted further optimisation using a Design of Experiments (DoE) strategy, which has been reported separately.^47^ The broader screening performed in our current work thus provides a valuable framework for identifying promising additive candidates and optimising synthesis conditions in future targeted studies. Compared to TEPA, the longer chain PEHA amine showed a lower percentage of faceted particles at the 1 : 100 ratio. This is possibly due to the amount of PEHA amine groups being too high from the combined effect of longer additive chain length and high additive concentration, thus causing a detrimental effect on the forming faceted particles. Overall, the increase in faceted particles present due to additive addition supports the hypothesis that the amines exhibit favourable binding *via* adsorption to the [111] face of the iron oxide.

What should also be taken into consideration is the flow within the systems used. While additives such as polyamines strongly influence final particle morphology,^31^ the flow environment provides a baseline level of control, enabling reproducibility and preventing the broad distributions typically seen in batch RTCP. Optimal flow rates were taken under consideration for the millifluidic flow system, however the continuous static mixer system did not explore multiple flow options. This is something to take under consideration moving forward with regards to optimising nanoparticle morphology and production yields.

Each system was operated successfully under the conditions described here, however fouling and clogging remain an important consideration for long-term continuous operation. In the static mixing system, precipitate build-up was observed within the Y-mixer and outlet tubing in under 90 s, requiring flushing between runs. The millifluidic reactor was somewhat more stable over extended operation, in agreement with previous studies (Norfolk *et al.* 2019 (ref. 26)), nonetheless some degree of fouling and leaking did occur. These observations highlight that while efficient mixing and nanoparticle formation are achievable, mitigation of fouling will be a factor to consider for future scale-up and sustained continuous production.

In addition, not only is throughput inherently limited in a millifluidic sheath flow system, but also the system renders itself unsuitable for scale-up since millimetric channels are required to ensure the required laminar flow. The highest production rate reported to date for MNPs has been 2.6 g h^−1^, or 62 g per day, using high temperature precipitation^35^ with the addition of surfactants.

In conclusion, using an additive-enhanced synthesis in a continuous static mixing system we demonstrated robust, scalable production of highly faceted iron oxide nanoparticles. The novelty presented here is the high theoretical productivity of the continuous static mixing system, reaching up to 311 g per day at laboratory scale, a fivefold increase over previously reported RTCP methods (see SI, Section S12). Efficient inline mixing, driven by static mixing elements, significantly reduced residence time without sacrificing product quality. This approach is inherently amenable to both scale-up and scale-out, facilitating translation of magnetite synthesis to continuous manufacturing at kilogram scale per day using green chemistry.

This versatile process provides an alternative, “greener” bioinspired additive method, where minute amounts of additives are needed, water is used as a solvent, and the reactions are all performed at room temperature. Recent studies have also begun incorporating static mixing into nanoparticle synthesis, including multistep modular systems and high-temperature surfactant-based routes,^50,52^ however the method described in this study achieves comparable control over particle morphology and magnetic properties using a simpler, greener, and ambient-temperature approach. This highlights the potential for continuous static mixers to not only scale SPION production but do so in a manner that aligns with environmentally sustain-able practices.

These findings establish continuous static mixing as a scalable and robust method for producing shape-controlled SPIONs under green RTCP conditions.

## Conflicts of interest

There are no conflicts to declare.

## Abbreviations

DETA: Diethylenetriamine
EDA: Ethylenediamine
MNP: Magnetic nanoparticle
MD: Molecular dynamics
PEEK: Polyether ether ketone
PEHA: Pentaethylenehexamine
RTCP: Room temperature co-precipitation
SPION: Superparamagnetic iron oxide nanoparticle
TEPA: Tetraethylenepentamine
TEM: Transmission electron microscope
TETA: Triethylenetetramine
VSM: Vibrating sample magnetometry
XRD: X-ray diffraction

## Supplementary Material

NA-OLF-D5NA00773A-s001

## Data Availability

Data supporting the findings of this study are openly available *via* Zenodo at https://doi.org/10.5281/zenodo.16779453. Supplementary information: Transmission Electron Microscopy (TEM) data, X-ray Diffraction (XRD) data, Vibrating Sample Magnetometry (VSM) data, supporting calculations, spreadsheets, and analyses related to nanoparticle size, morphology, and magnetic properties. All files are licensed under CC-BY 4.0. See DOI: https://doi.org/10.1039/d5na00773a.
