# Supplementary material for: Enhanced control and production rates for a green continuous flow synthesis of magnetite nanoparticles: a comparative study of ethylenediamine additives
Source: Nanoscale Adv. 2025 Oct 15;7(23):7798–810. doi: 10.1039/d5na00773a (PMC12538300; doi:10.1039/d5na00773a)
Supplement: NA-007-D5NA00773A-s001 [file NA-007-D5NA00773A-s001.pdf]

Supplementary Information (SI) for Nanoscale Advances.
This journal is © The Royal Society of Chemistry 2025

Enhanced Control and Production Rates for a Green Continuous Flow Synthesis of Magnetite Nanoparticles: A Comparative Study of Ethylenediamine Additives†

Georgina Zimbitas[a◊], Laura Norfolk[b◊], Jan Sefcik[c], Sarah Staniland[*b‡]

[a]Faculty of Medicine, Health, & Social Care, Canterbury Christ Church University, Canterbury CT1 1QU, UK
[b]Department of Chemistry, The University of Sheffield, Sheffield S3 7HF, UK. E-mail: S.S.Staniland@sheffield.ac.uk
[c]Department of Chemical & Process Engineering, University of Strathclyde, Glasgow G1 1XJ, UK
◊ joint first authors
‡Present address: Government Office for Technology Transfer, UK Government

SUPPLEMENTARY INFO

S1. Particle Sizing Methodology

ImageJ software was used to measure particles. In order to ensure consistency, all measurements were done across the longest axis of each particle. As there is a variety in particles present, approximately 200 measurements are taken per sample to obtain a reasonable mean size. A single "area" of the TEM image is selected for the analysis. This is done to avoid human bias, e.g., by avoiding the chance of larger (or smaller) particles being selectively measured.

Figure S1: Screenshot of ImageJ particle sizing, showing particles analysed in clusters. Image reproduced from Supplementary Information included in Norfolk et al., Ethylenediamine series as additives to control the morphology of magnetite nanoparticles, Green Chemistry, 23, 5724-5735 (2021).

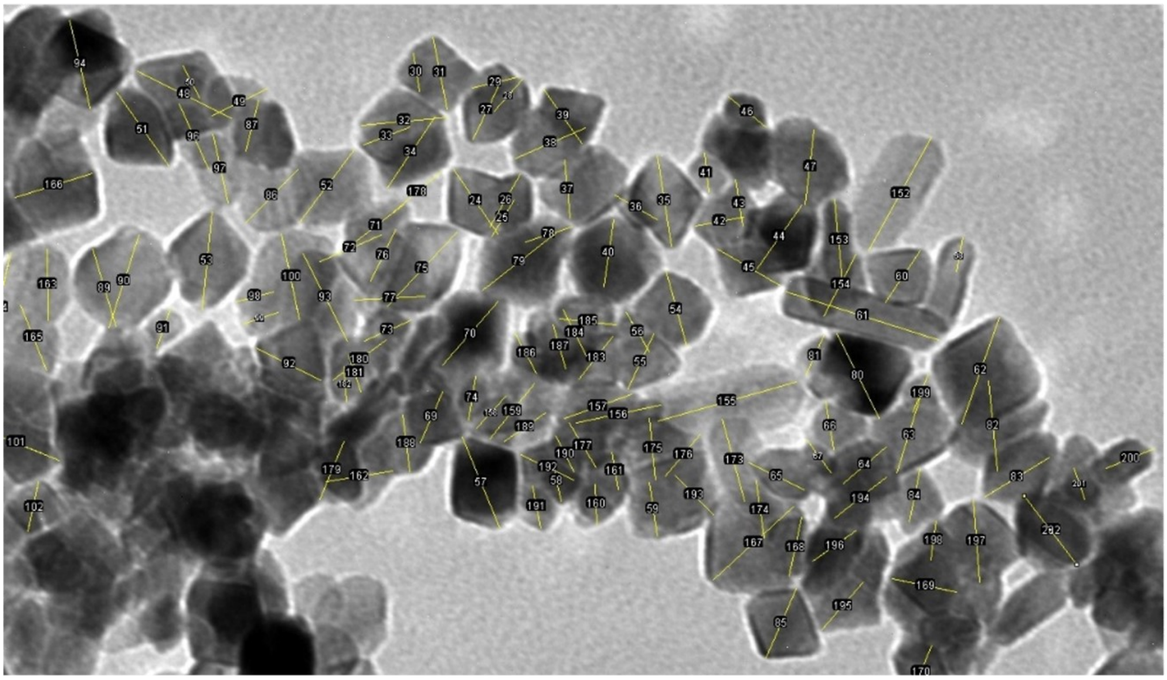

S2. Particle Shape Analysis Methodology

Particles were assessed manually from TEM images. Manual analysis of images is done using drawing software (Inkscape, Paint.net, etc.). Each particle is individually marked for shape with a set colour (figure S3). For each sample there are several images analysed to ensure that a minimum of 300 particles are marked. Particles with clearly visible flat crystal faces were classified as faceted and include those that appeared cubic, octahedral, or similar. Assignment of specific morphology could not be made due to the fact that particle orientation within the 2D image could lead to misinterpretation. Particles that overlapped with others or were oriented in such a way that faceting could not be confidently identified were classified as unidentified. Classification criteria were consistent as applied across all images. In order to maximise accuracy, shape assignment is performed again once all assigned particles have been counted. An indication of how sample particle shapes are assigned can be seen in Figure S2.

Figure S2: Example image analysis showing faceted (octahedral/square: red, hexagonal: purple), and undefined (green), particles. Coloured boxes highlight identified faceted particles. Image reproduced from Supplementary Information included in Norfolk et al., Ethylenediamine series as additives to control the morphology of magnetite nanoparticles, Green Chemistry, 23, 5724-5735 (2021).

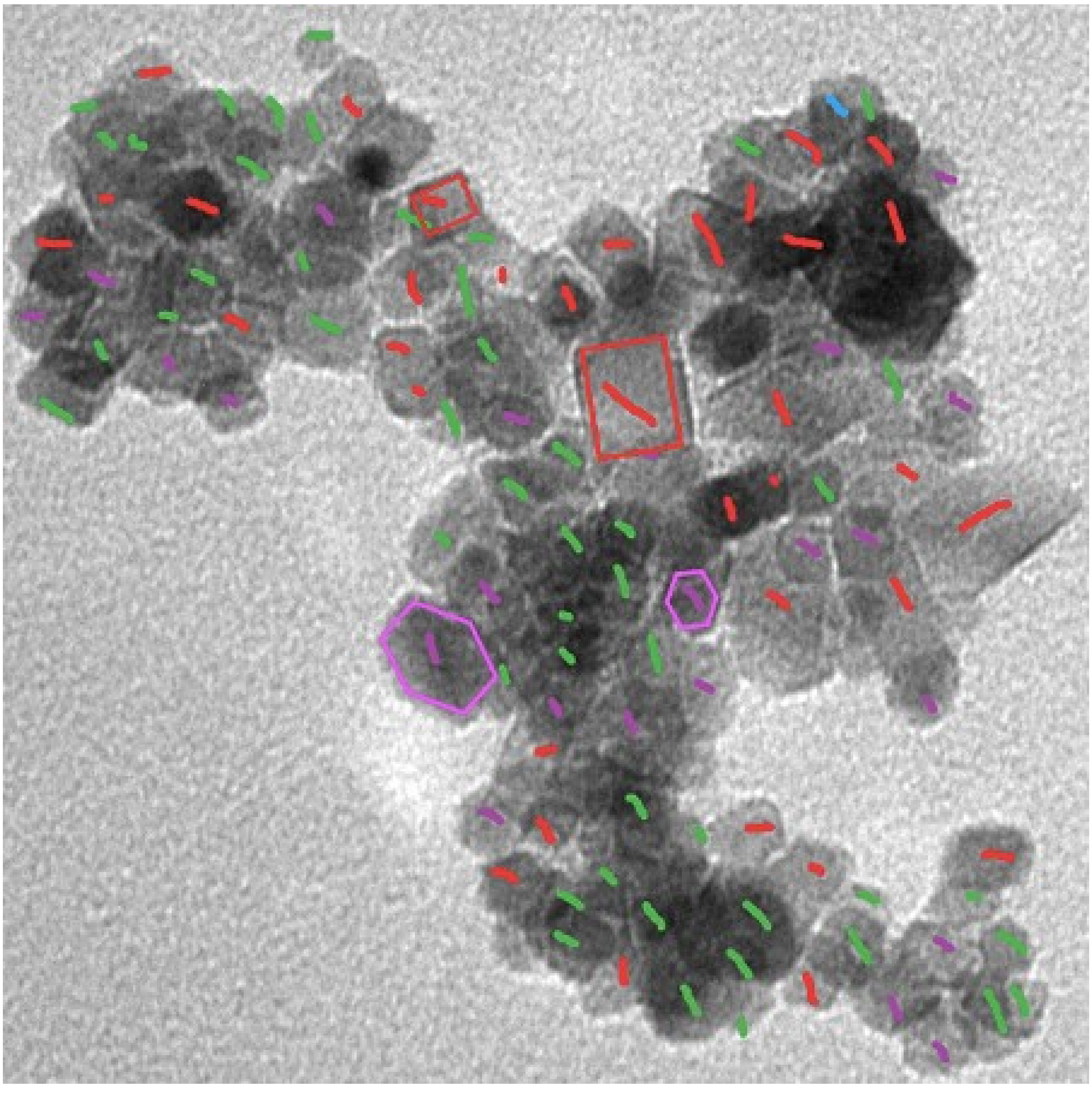

S3. XRD data for control (no additive present) sample

Below are the XRD spectra for the control (no additive) of each of the 3 setups described in the paper.

Figure S3: XRD data of control (no additive) RTCP reactions for each set-up system: batch RTCP (red), millifluidic flow (green), and continuous static mixing (blue). Impurities present in some samples may be due to some degree of oxidation having occurred prior to XRD analysis. These impurities appear to be minor and as such are not considered to affect the overall results dramatically.

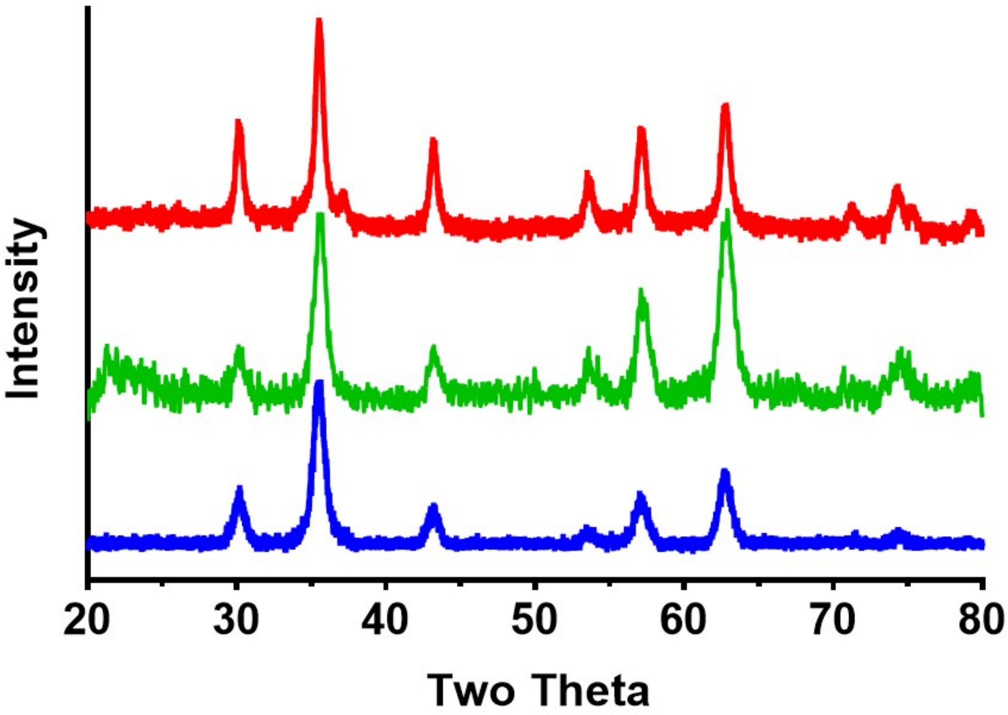

S4. Results for each ethylenediamine sample.

Results presented separately.

Figure S4: EDA additive data compilation: a) i-vi. TEM images; b) shape analysis; c) frequency distribution; d) XRD; e) magnetic data of particles formed at 1:100 (pale red), 1:1000 (red) and 1:10000 (dark red) EDA:iron ion ratios Solid lines indicate additive added through Fe inlet, and dashed lines indicate through NaOH inlet.

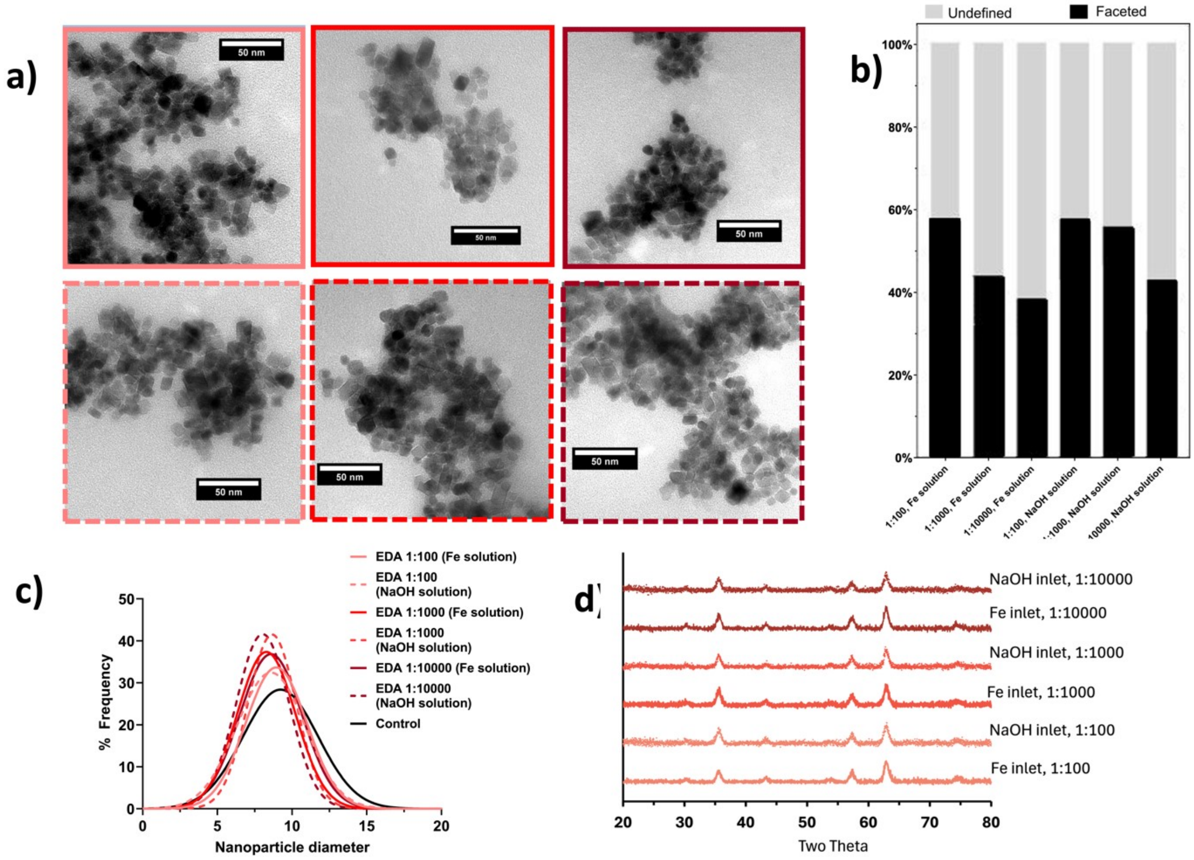

S5. Results for each DETA sample.

Results presented separately.

Figure S5: a) DETA additive data compilation: i-vi. TEM images; b) shape analysis; c) frequency distribution; d) XRD; e) magnetic data of particles formed at 1:100 (pale green), 1:1000 (green) and 1:10000 (dark green) DETA:iron ion ratios Solid lines indicate additive added through Fe inlet, and dashed lines indicate through NaOH inlet.

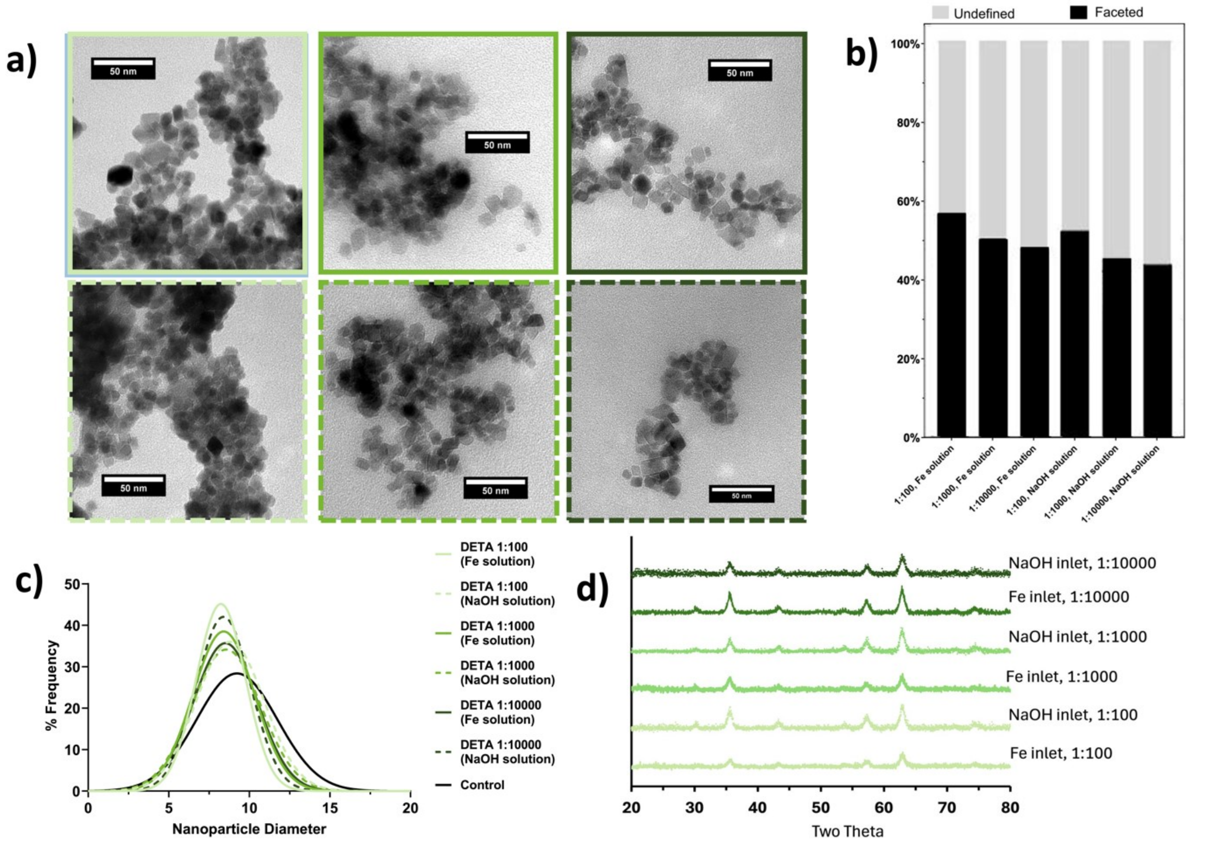

S6. Results for each TETA sample.

Results presented separately.

Figure S6: TETA additive date compilation: a) TEM images; b) shape analysis; c) frequency distribution; d) XRD; e) magnetic data of particles formed at 1:100 (light blue), 1:1000 (blue) and 1:10000 (dark blue) TETA:iron ion ratios Solid lines indicate additive added through Fe inlet, and dashed lines indicate through NaOH inlet.

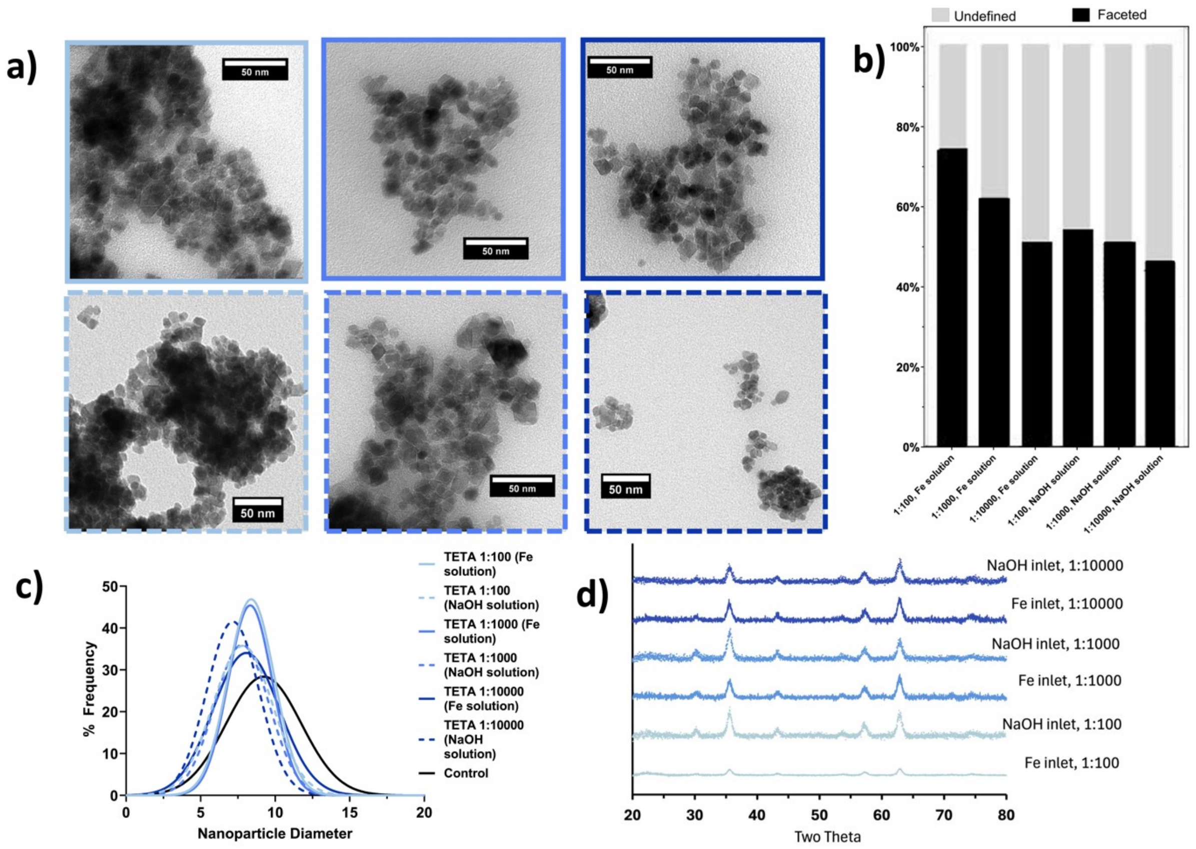

S7. Results for each TEPA samples.

Results presented separately.

Figure S7: TEPA additive data compilation: a) TEM images; b) shape analysis; c) frequency distribution; d) XRD; e) magnetic data of particles formed at 1:100 (pale purple), 1:1000 (purple) and 1:10000 (dark purple) TEPA:iron ion ratios Solid lines indicate additive added through Fe inlet, and dashed lines indicate through NaOH inlet.

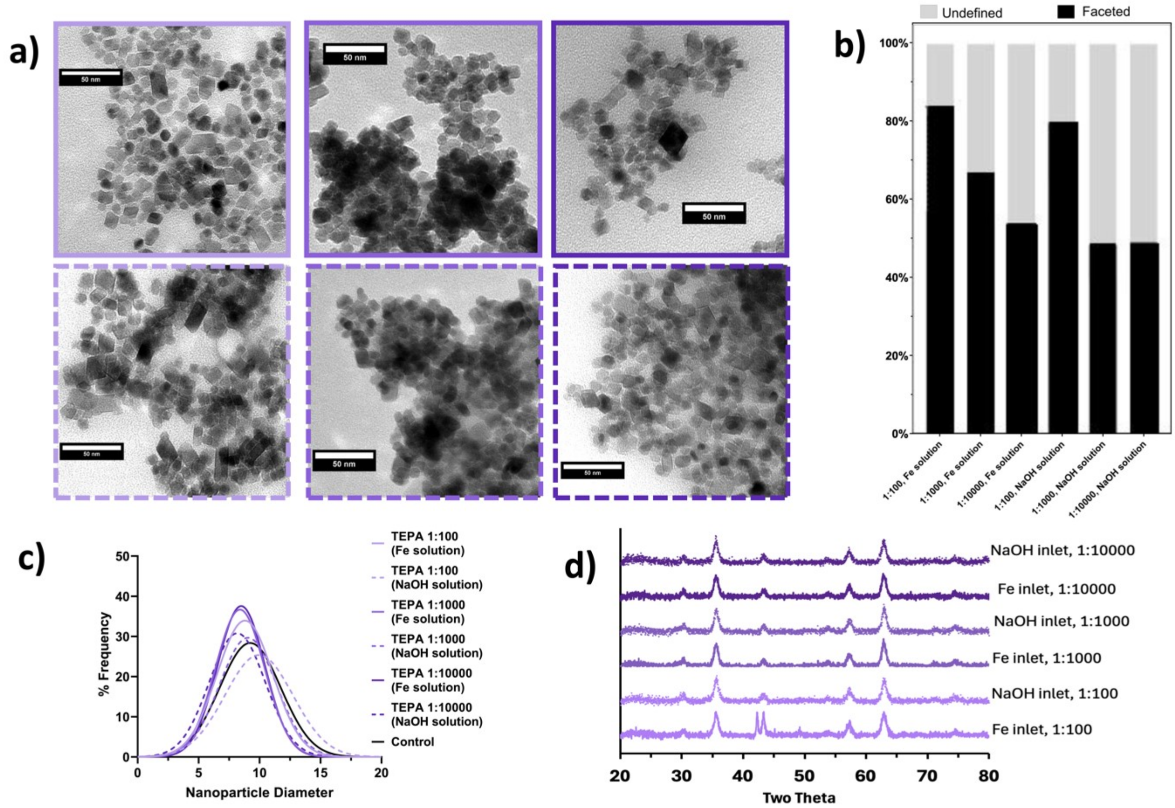

S8. Results for each PEHA samples.

Results presented separately.

Figure S8: PEHA additive data compilation: a) TEM images; b) shape analysis; c) frequency distribution; d) XRD; e) magnetic data of particles formed at 1:100 (lighter pink), 1:1000 (pink) and 1:10000 (darker pink) PEHA:iron ion ratios. Solid lines indicate additive added through Fe inlet, and dashed lines indicate additive added through NaOH inlet.

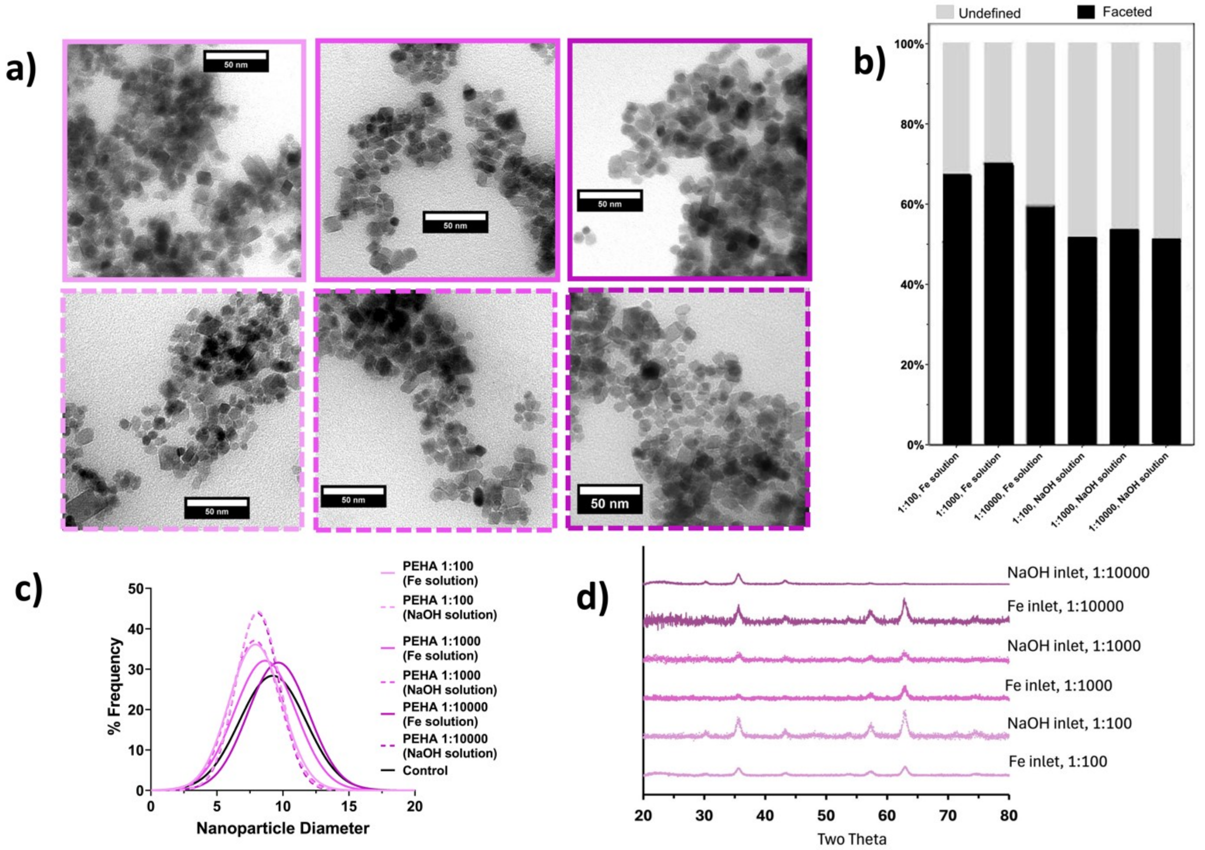

S9. Percentage of faceted particles produced in each additive compilation

Figure S9: Graphs showing percentage faceted particles against additive:iron ratio for EDA (red), DETA (green), TETA (blue), TEPA (purple), and PEHA (pink)

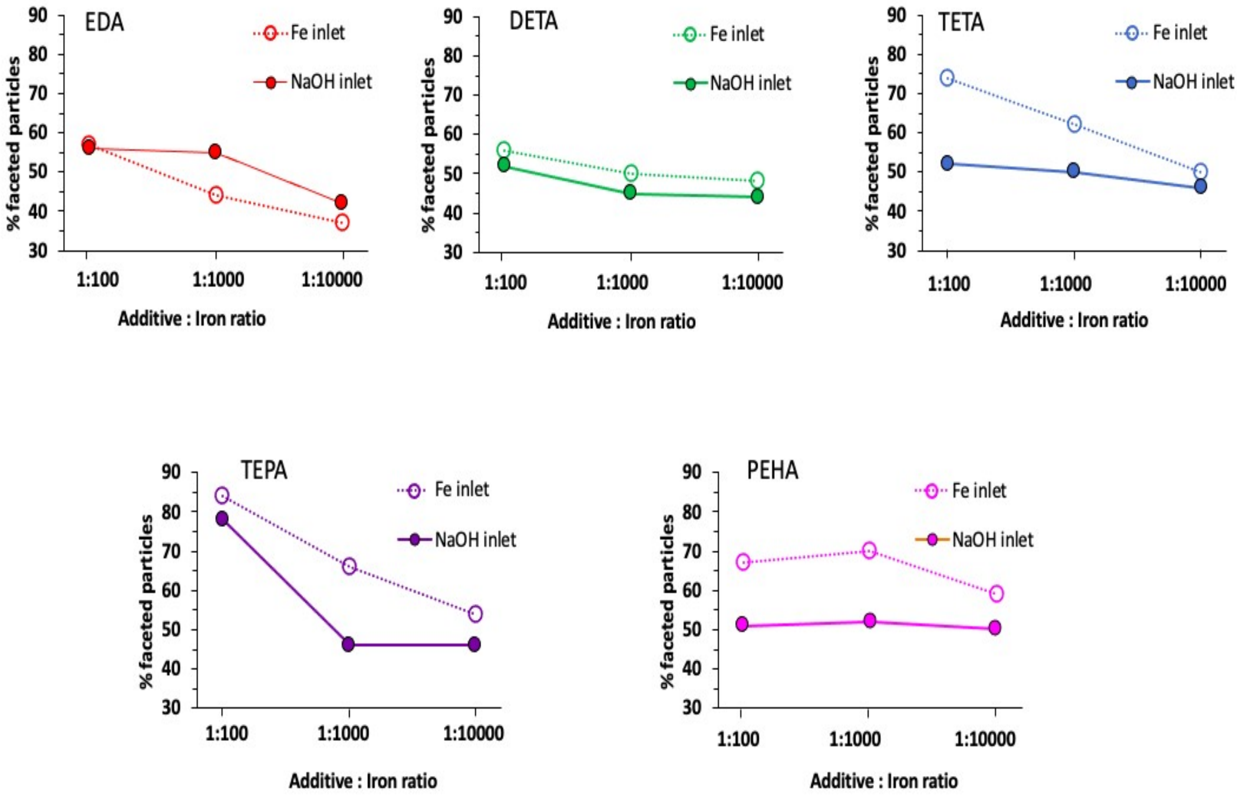

S10. Saturation magnetisation for the products of each additive compilation

Figure S10: Saturation magnetisation against additive:iron ratio for EDA-PEHA additives added through a) Fe inlet and b) NaOH inlet.

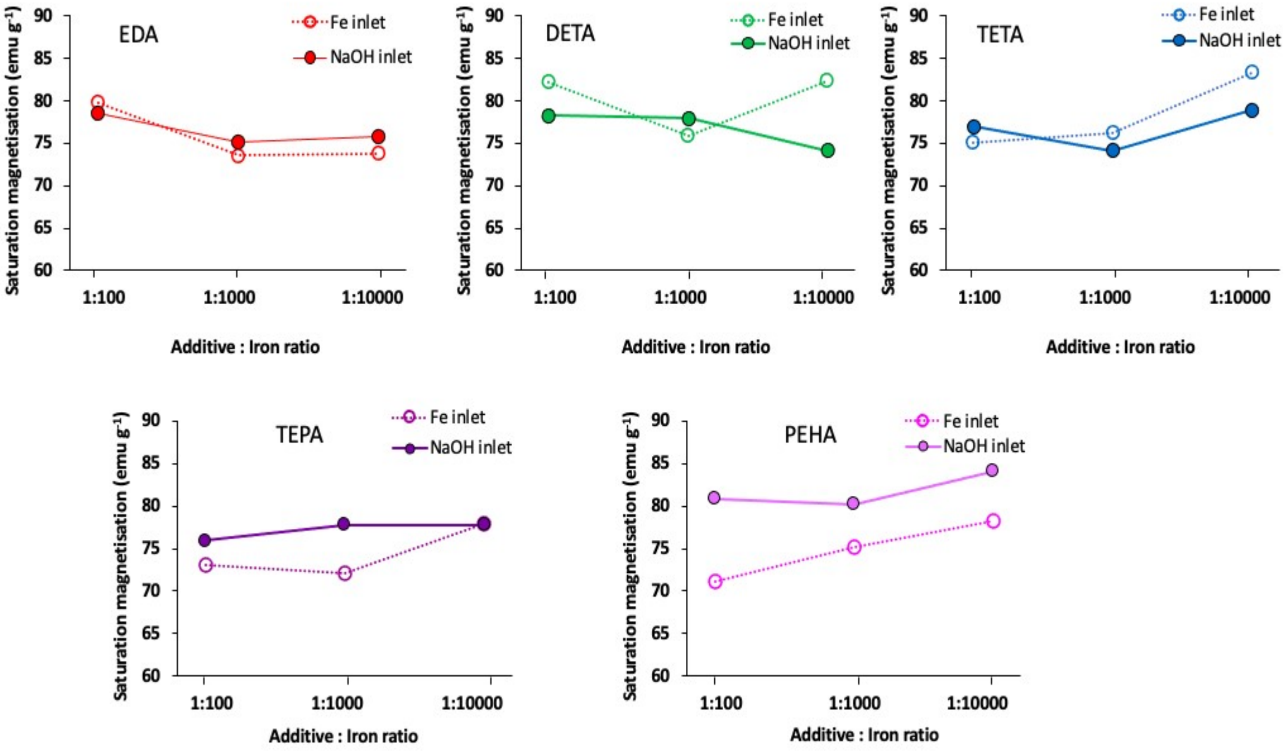

S11. Mean particle size for the products of each additive compilation

Figure S11: Graphs showing mean particle size against additive:iron ratio for EDA (red), DETA (green), TETA (blue), TEPA (purple), and PEHA (pink). Range bars show minimum and maximum particle size with the distributions shown in S4-S8.

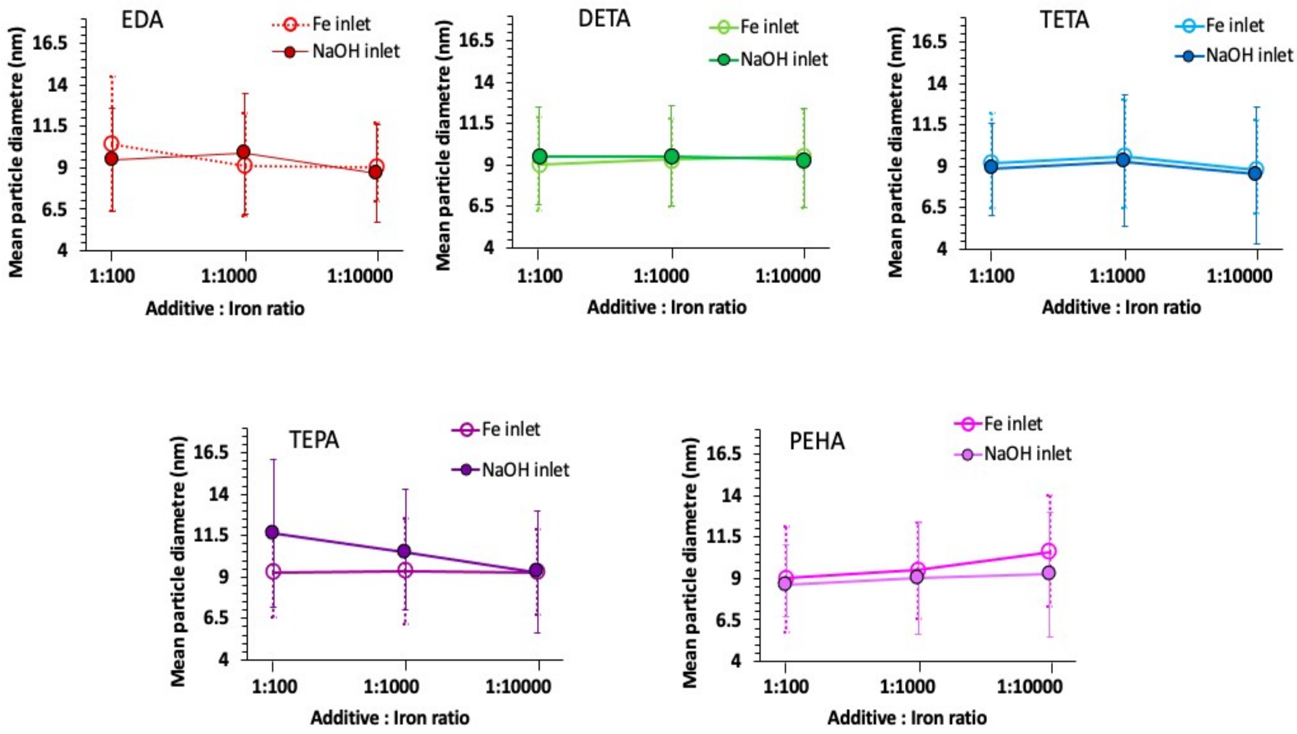

S12. Theoretical Production rate of magnetite

Theoretical production rate (g/day of nanoparticles) calculation. Assuming a 100% conversion rate of the precursor and including only magnetically separated material, theoretical production rates were calculated for the three systems run over a 24-hour period.

Reaction information used:

- Batch RTCP: 1 mmol of iron salts produces 0.333 mmol of magnetite per reaction
- Millifluidic flow: 90 µL/min flow rate of 50 mmol iron solution
- Continuous static mixing: Average flow rate of 112 mL/min (from 31 measurements) for both NaOH and Fe solution. As they flow in a 1:1 ratio the flow rate of only the Fe solution can be assumed to be half the overall flow rate - 56 mL/min

Assumptions:

1. Time for system set-up and particle extraction from the batch system between batches is not accounted for
2. All batch RTCP synthesis is run directly after one another for a 24-hour period (9 batches)
3. Millifluidic flow and continuous static mixing systems run continuously for a 24-hour period
4. 100% conversion of iron salt to magnetite (Fe_3O_4 molecular weight = 231.531 g/mol, 3 mol of iron salt produces 1 mol of magnetite)

Calculations:

Batch RTCP: 3×10^{-3} mol of magnetite x 231.531 g/mol = 0.69 g/day

Millifluidic flow: 1440 (min/day) x 90 µL/min = 0.1296 L/day 0.1296 L x (0.05/3) Mol of magnetite formed x 231.531 g/ mol = 0.50 g/day

Continuous static mixer: 1440 (mins/day) x 56 mL/min = 80.64 L/day of iron solution processed 80.64 L x (0.05/3) Mol of magnetite formed x 231.531 g/mol= 311.2 g/day
